# Supplementary material for: KG-Hub—building and exchanging biological knowledge graphs
Source: Bioinformatics. 2023 Jun 30;39(7):btad418. doi: 10.1093/bioinformatics/btad418 (PMC10336030; doi:10.1093/bioinformatics/btad418)
Supplement: btad418_Supplementary_Data [file btad418_supplementary_data.docx]

| **Component** | **Purpose** | **Source code** | **Website** | **External project?** |
| --- | --- | --- | --- | --- |
| kghub.org | Main website | https://github.com/Knowledge-Graph-Hub/knowledge-graph-hub.github.io | kghub.org |  |
| kg-hub.berkeleybop.io/ | Browsing kg-hub graph data and other artifacts | - | https://kg-hub.berkeleybop.io/ |  |
| kghub-downloader | Downloading data from remote file and APIs | https://github.com/monarch-initiative/kghub-downloader | - |  |
| Koza | Easy, declarative transform of data into graph form, validation | https://github.com/monarch-initiative/koza | - |  |
| KG-Hub example repository | Exemplar KG-Hub project | https://github.com/Knowledge-Graph-Hub/kg-example | - |  |
| KG-Hub cookie-cutter template | Template for starting a new KG-Hub project | https://github.com/Knowledge-Graph-Hub/kg-cookiecutter | - |  |
| KGX | Converting and manipulating graph data | https://github.com/biolink/kgx | - |  |
| GRAPE | Fast, performant graph machine learning | https://github.com/AnacletoLAB/grape | n/a |  |
| Biolink Model | High-level data model of biological data, to facilitate interoperability of KGs, implemented in LinkML | https://github.com/biolink/biolink-model | https://biolink.github.io/biolink-model/ |  |
| LinkML | Modeling Language for linked data | https://github.com/linkml/linkml | https://linkml.io |  |
| Robot | Software for automating ontology development tasks | https://github.com/ontodev/robot | http://robot.obolibrary.org/ |  |
| Dashboard | Visual display of data contained in each KG-Hub project | https://github.com/Knowledge-Graph-Hub/kg-hub-dashboard | https://kghub.org/kg-hub-dashboard/ |  |
| Blazegraph Runner | Generation of blazegraph journal | https://github.com/balhoff/blazegraph-runner | - |  |
| NEAT | Declarative language for describing and running graph ML tasks using GRAPE | https://github.com/Knowledge-Graph-Hub/neat-ml | - |  |
| KG-Hub tutorials | Jupyter notebook tutorials for creating and using KG-Hub graph | https://github.com/Knowledge-Graph-Hub/knowledge-graph-hub-support/tree/main/kg-hub-tutorials | - |  |
| KG-Hub support page | Collection of resources for issues, support, and discussion | https://github.com/Knowledge-Graph-Hub/knowledge-graph-hub-support | - |  |
| Jenkins | Continuous integration/continuous deployment of graph building pipeline | https://github.com/jenkinsci | https://www.jenkins.io/ | ✔️ |
| Github | Source code management of ETL and other code | github.com | github.com | ✔️ |
| Amazon S3 | Cloud storage of graph artifacts | - | https://aws.amazon.com/s3/ | ✔️ |
| Amazon EC2 | Used for automated graph ML tasks described by NEAT | - | https://aws.amazon.com/ec2/ | ✔️ |
| Neo4j | Graph database, to which KG-Hub projects optionally can export | - | https://neo4j.com/ | ✔️ |
| Blazegraph | Graph database, to which KG-Hub projects optionally can export | - | https://blazegraph.com/ | ✔️ |

Supplementary Table 1. Internal and external software components of KG-Hub. A description of the purpose of each component and links to the source code and website (if applicable) are shown.


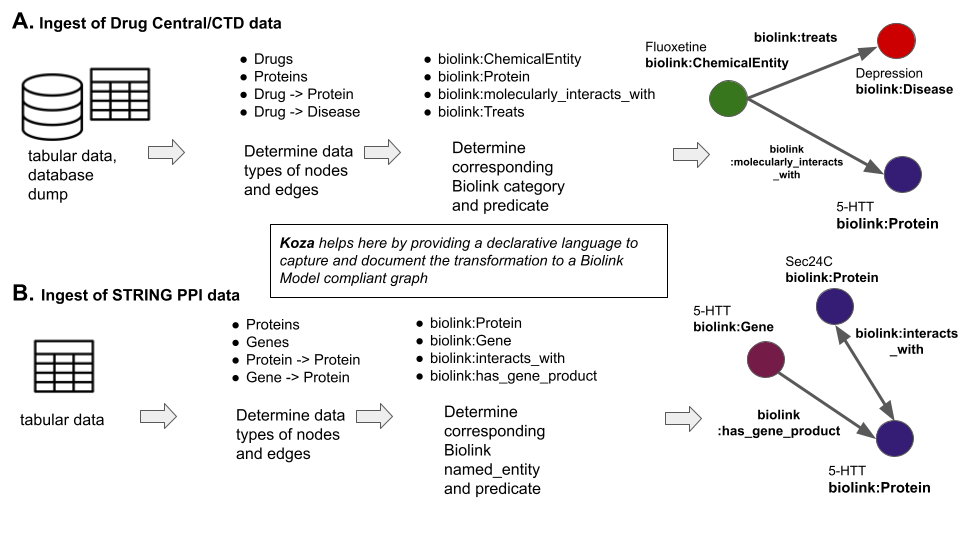


Supplemental Figure 1. Biolink Model example use case showing the process of data harmonization from two ingests. A. Data from Drug Central to be ingested into the KG (e.g. drug -> protein links via <https://unmtid-shinyapps.net/download/DrugCentral/2021_09_01/drug.target.interaction.tsv.gz> and drug to disease via CTD (The Comparative Toxicogenomic Database) <http://ctdbase.org/>) are identified. The data types of all nodes (e.g. protein, drugs, etc) and edges (e.g. drug to protein associations) are determined through a process of manual data reconciliation facilitated by the annotations, computable mappings, translation tables, definitions, identifier mappings, and implementation documentation (e.g. <https://biolink.github.io/biolink-model/guidelines/reading-a-qualifier-based-statement.html>) provided by Biolink Model. Koza provides a declarative and computable language to capture and document the transformation to a Biolink Model compliant graph. Importantly, changes in the documentation or use cases for transformation are automatically reflected in the code, keeping the subject matter expertise used to transform a source to be Biolink Model compliant close to the code that executes the transform. Knowledge graphs built using Koza will have immediate access to these curated transformations and can reuse the work done to transform external data sources in a consistent manner. In addition, because of the active community of Biolink Model developers, when a particular class or attribute is not available or ambiguous in the model, the model is updated and or extended (and tagged and released using semantic versioning). The modelers take into account the use case of interest as well as the conventions in the model while extending the model. Because Biolink Model is a living standard, use case reconciliation, and extension of the model happens frequently and contributes to the useability of the model for the community.

Target:

target_path: output_data

GraphDataConfiguration:

graph:

directed: False

node_path: ./merged-kg_nodes.tsv

edge_path: ./merged-kg_edges.tsv

verbose: True

nodes_column: id

node_list_node_types_column: category

default_node_type: biolink:NamedThing

sources_column: subject

destinations_column: object

default_edge_type: biolink:related_to

EmbeddingsConfig:

filename: embedding.csv

history_filename: embedding_history.json

node_embeddings_params:

method_name: "First-order LINE"

iterations: 20

tsne_filename: tsne.png

ClassifierContainer:

classifiers:

- classifier_id: lr_1

classifier_name: Logistic Regression

classifier_type: sklearn.linear_model.LogisticRegression

edge_method: Average

outfile: "model_lr_out"

parameters:

sklearn_params:

random_state: 42

max_iter: 100

ApplyTrainedModelsContainer:

models:

- model_id: lr_1

node_types:

source:

- "biolink:Protein"

destination:

- "biolink:Protein"

cutoff: 0.5

outfile: lr_protein_predictions.tsv

Upload:

s3_bucket: your-favorite-cloud-storage

s3_bucket_dir: target_directory

extra_args:

"ACL": public-read

Supplemental Figure 2. An example YAML file used to produce node embeddings and edge predictions with a KG-Hub graph for use in ML analysis via the NEAT package. This configuration also instructs NEAT to upload all outputs to a directory in an AWS S3 bucket and make the objects publicly viewable.

| **Filename** | **Contents** |
| --- | --- |
| download.yaml | ---  # Three selected citations from PubMed  -  url: https://eutils.ncbi.nlm.nih.gov/entrez/eutils/esummary.fcgi?db=pubmed&id=26539902,7037108,27605131  local_name: docs.xml  # Three selected proteins from UniProtKB  -  url: https://www.ebi.ac.uk/proteins/api/proteins?offset=0&size=100&accession=Q257X2%2CP13296%2CP0DN38  local_name: proteins.xml |
| pubmed.yaml | name: 'pubmed_transform'  files:  - './docs.json'  format: 'json'  node_properties:  - 'id'  - 'name'  - 'category'  transform_mode: 'flat' |
| pubmed.py | from biolink.model import Publication  from koza.cli_runner import get_koza_app  koza_app = get_koza_app("pubmed_transform")  row = koza_app.get_row()  pub_id = "PUBMED:" + row["Id"]  for item in row["Item"]:  if item["@Name"] == "Title":  name = item["#text"]  pub = Publication(  id=pub_id,  name=name,  publication_type="dc:type",  category=["biolink:Publication"]  )  koza_app.write(pub) |
| uniprot.yaml | name: 'uniprot_transform'  files:  - './proteins.json'  format: 'json'  node_properties:  - 'id'  - 'name'  - 'category'  edge_properties:  - 'id'  - 'subject'  - 'predicate'  - 'object'  - 'category'  transform_mode: 'flat' |
| uniprot.py | import uuid  from biolink.model import Association, Protein, OrganismTaxon  from koza.cli_runner import get_koza_app  koza_app = get_koza_app("uniprot_transform")  row = koza_app.get_row()  try:  SPECIES_SET == SPECIES_SET  except NameError:  SPECIES_SET = []  if isinstance(row["accession"], list):  prot_id = "UniProtKB:" + row["accession"][0]  else:  prot_id = "UniProtKB:" + row["accession"]  name = row["name"]  prot = Protein(  id=prot_id,  name=name,  category=["biolink:Protein"]  )  taxid = "NCBITaxon:" + row["organism"]["dbReference"]["@id"]  if taxid not in SPECIES_SET:  SPECIES_SET.append(taxid)  for name in row["organism"]["name"]:  if name["@type"] == "scientific":  taxname = name["#text"]  taxon = OrganismTaxon(  id=taxid,  name=taxname,  category=["biolink:OrganismTaxon"]  )  koza_app.write(taxon)  association = Association(  id="uuid:" + str(uuid.uuid1()),  subject = prot.id,  predicate = "biolink:in_taxon",  object = taxon.id  )  koza_app.write(association) |
| run.sh | pip install biolink-model kghub-downloader koza yq  downloader  cat docs.xml \| xq '.eSummaryResult.DocSum' > docs.json  cat proteins.xml \| xq '.uniprot.entry' > proteins.json  koza transform --source pubmed.yaml  koza transform --source uniprot.yaml |

Supplementary Table 2. Example code and instructions for data download and transformation in a KG-Hub project. On a Linux system, a directory containing the files named above and their respective contents is sufficient to run this process; it may be run with *./run.sh*. For brevity, filenames do not follow KG-Hub project layout conventions; this is a minimal working example. The example uses the kghub-downloader module, the *xq* module of the *jq* tool (provided through the *yq* Python module), and the Koza package to retrieve XML data, do an initial preprocessing transform, and a subsequent transform to represent it in KGX TSV format, respectively. The data sources are PubMed citations and UniProtKB protein records. The *jq* tool must be installed beforehand: see <https://stedolan.github.io/jq/> for detailed instructions.
